# Nanotube breakthroughs: unveiling the potential of carbon nanotubes as a dual therapeutic arsenal for Alzheimer’s disease and brain tumors

**DOI:** 10.3389/fonc.2023.1265347

**Published:** 2023-09-20

**Authors:** Deena Elsori, Gowhar Rashid, Nihad Ashraf Khan, Punya Sachdeva, Riya Jindal, Falak Kayenat, Bhuvi Sachdeva, Mohammad Azhar Kamal, Asaad Ma Babker, Sherif Ashraf Fahmy

**Affiliations:** ^1^Faculty of Resillience, Deans Office Rabdan Academy, Abu Dhabi, United Arab Emirates; ^2^Amity Medical School, Amity University Gurgaon, Haryana, India; ^3^Department of Biosciences, Faculty of Natural Sciences, Jamia Millia Islamia, New Delhi, India; ^4^Department of Neuropyschology and Neurosciences, Amity University, Noida, UP, India; ^5^Department of Biotechnology, Shoolini University, Himachal Pradesh, India; ^6^Department of Biotechnology, Jamia Hamdard University, New Delhi, India; ^7^Department of Physics and Astrophysics, Bhagini Nivedita College, University of Delhi, New Delhi, India; ^8^Department of Pharmaceutics, College of Pharmacy, Prince Sattam Bin Abdulaziz University, Alkharj, Saudi Arabia; ^9^Department of Medical Laboratory Sciences, Gulf Medical University, Ajman, United Arab Emirates; ^10^Department of Chemistry, School of Life and Medical Sciences, University of Hertfordshire Hosted by Global Academic Foundation, Cairo, Egypt

**Keywords:** nanotubes, nanoparticles, Alzheimer’s disease, carbon nanotubes, brain tumor

## Abstract

Alzheimer’s disease (AD) and brain tumors are debilitating neurological conditions that pose significant challenges in current medical practices. Existing treatment options for AD primarily focus on symptom management, and brain tumors often require aggressive therapeutic approaches. Novel disease-modifying strategies and therapeutic agents are urgently needed to address the underlying causes of AD pathogenesis and improve brain tumor management. In recent years, nanoparticles (NPs) have shown promise as valuable tools in diagnosing and managing various brain disorders, including AD. Among these, carbon nanotubes (CNTs) have garnered attention for their unique properties and biomedical potential. Their ability to cross the blood-brain barrier (BBB) with ease opens up new possibilities for targeted drug delivery and neuroprotection. This literature review aims to explore the versatile nature of CNTs, which can be functionalized with various biomolecules or substances due to their sp2 hybridization. This adaptability enables them to specifically target cells and deliver medications under specific environmental conditions. Moreover, CNTs possess an exceptional capacity to penetrate cell membranes, making them valuable tools in the treatment of AD and brain tumors. By delving into the role of CNTs in biomedicine, this review sheds light on their potential in managing AD, offering a glimpse of hope for effective disease-modifying options. Understanding the mechanisms of CNTs’ action and their capabilities in targeting and delivering medication to affected cells will pave the way for innovative therapeutic strategies that can improve the lives of those afflicted with these devastating neurological conditions. The exploration of CNTs as a dual therapeutic arsenal for both brain tumors and Alzheimer’s disease holds great promise and may usher in a new era of effective treatment strategies for these challenging conditions.

## 1 Introduction

Alzheimer’s disease (AD) and brain tumors represent two of the most complex and devastating neurological conditions, affecting millions worldwide (1, 2). Despite significant research efforts, effective disease-modifying treatments for both AD and brain tumors remain elusive (3). Current approaches often focus on managing symptoms rather than addressing the underlying causes, burdening patients and their families with the progressive deterioration of cognitive function or the challenges of aggressive tumor growth (4).

In recent years, nanotechnology has emerged as a promising frontier in biomedical research, offering potential solutions to various brain disorders (5). Among these nanomaterials, carbon nanotubes (CNTs) have garnered substantial attention for their exceptional properties and biomedical versatility (6). With their unique ability to easily traverse the blood-brain barrier (BBB), CNTs open new avenues for targeted drug delivery and neuroprotection in the intricate environment of the brain (7).

CNTs possess remarkable properties stemming from their sp2 hybridization, allowing them to be functionalized with various biomolecules and substances (8). This adaptability enables CNTs to specifically target cells and deliver therapeutic agents precisely where needed, even under specific environmental conditions. Additionally, CNTs’ exceptional capacity to penetrate cell membranes makes them potential game-changers in treating both AD and brain tumors (9).

CNTs have several advantages as compared to other nanosystems, such as unique physicochemical properties, biological interaction with brain cancerous tissues, ability to penetrate BBB, and bio-corona effect (10).

In this literature review, we aim to explore the transformative potential of CNTs as a dual therapeutic arsenal for both brain tumors and Alzheimer’s disease. By comprehending the intricacies of CNTs’ applications in biomedicine, we seek to shed light on their role in neuroprotection and targeted medication delivery. Understanding the mechanisms of CNTs’ action and their capabilities in targeting affected cells holds promise for devising innovative and effective disease-modifying strategies.

As the search for more sustainable and potent treatments for AD and brain tumors intensifies, exploring CNTs as a therapeutic tool provides hope for groundbreaking advancements in neurology and oncology. Our quest to unveil the true potential of carbon nanotubes brings us closer to addressing the challenges posed by these devastating neurological conditions, offering a glimmer of hope to those affected and their loved ones.

**Figure 1:**
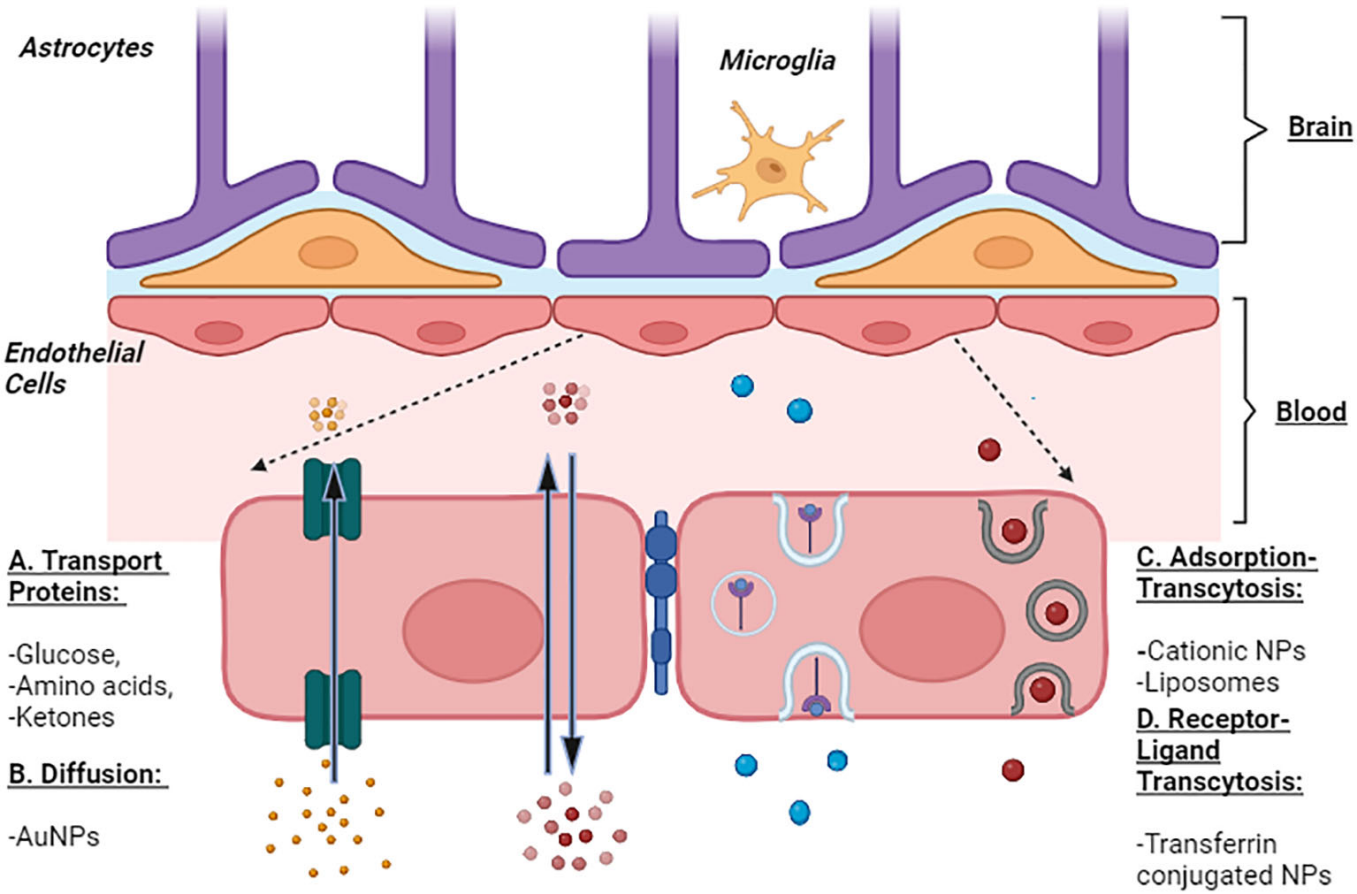
Representation of Blood Brain Barrier and Transport of Nanoparticles. The transport of NPs across BBB is represented by four prominent mechanisms commonly enrooted for the transport of solute molecules. A: Transport Proteins - these channel proteins facilitate the passage of glucose, amino acids, and ketones across the BBB and hence, can be used to transport NPs. B: Diffusion - passage of molecules from higher concentration to lower such as small lipophilic molecules, O2, and AuNPs. C: Adsorption-mediated Transcytosis - several cationic molecules, liposomes, and cationic NPs are endocytosed across BBB. D: Receptor-mediated transcytosis is facilitated by receptor-ligand interaction as seen in the transport of iron mediated by transferrin (receptor). NPs can be transported along this mechanism via the transferrin NPs conjugates.

The types of nanoparticles (NPs) can be transported are shown in (**Figure 1**).

Carbon nanotubes (CNTs), one of the types of NPs, are a potent tool for enhancing biomedical methods in treating and managing many diseases, including AD and Brain malignancies (11, 12). CNTs can adsorb different molecules of drugs on their surface (13). CNTs have good electronic properties, significant capability to cross the cell membrane and BBB, thermal properties, high drug loading capacity, and can be easily modified with other molecules (9). In this literature review, we discuss the role of CNTs as a tool for delivering drugs or chemical compounds to the brain.

## 2 Nanoparticles: an approach to manage AD and brain tumor

Nanoparticles have revolutionized medicine, offering new and promising possibilities for managing challenging conditions like Alzheimer’s disease and brain tumors. Their unique properties, particularly their small size, make them excellent candidates for targeted drug delivery and therapeutic interventions, allowing precise treatment targeting specific areas, such as the brain (14). In this context, **Table 1** presents a comprehensive overview of diverse nanoparticle types that have been extensively investigated in the pursuit of effective Alzheimer’s disease management and Brain tumor.

**Table 1:** Comparative analysis of nanoparticle-based approaches for alzheimer’s disease and brain tumor.

Nanoparticle Type	Application in Alzheimer’s Disease	Application in Brain Tumors
Lipid-based Nanoparticles	Delivering anti-amyloid drugs to the brain to reduce amyloid plagues and prevent disease progression (15)	Targeted delivery of chemotherapeutic agents, reducing systemic toxicity and enhancing drug accumulation in tumor tissues(16)
Polymeric Nanoparticles	Encapsulating neuroprotective agents to shield neurons from degeneration and improve cognitive function (17).	Delivering gene therapy to brain tumor cells interferes with tumor growth and promotespoptosis (18).
Metal-based Nanoparticles	Binding to amyloid beta peptides and facilitating their clearance from the brain, potentially slowing down Alzheimer’s progression (15).	Acting as a contrast agent in imaging techniques for brain tumor detection and monitoring (19).
Mesoporous Silica Nanoparticles	Loading and releasing therapeutic molecules in a controlled manner, offering sustained drug release for Alzheimer’s treatment (20).	Delivering a combination of therapeutic agents for synergistic effects in brain tumor therapy (21).
Dendrimers	Enhancing drug solubility and stability, improving drug delivery efficiency in Alzheimer’s disease (22).	Penetrating the blood-brain barrier (BBB) and delivering a variety of therapeutics, including chemotherapy drugs and immunotherapies (23).
Carbon Nanotubes	Facilitating targeted drug delivery by functionalization with ligands that bind to specific receptors in Alzheimer’s-affected brain region (24).	Acting as nanocarriers for brain tumor-targeting agents, such as small interfering RNA (siRNA) for gene silencing (25).
Nanogels	Formulating hydrogel-based nanoparticles for sustained drug release and increased therapeutic efficacy in Alzheimer’s treatment (26).	Encapsulating anticancer drugs for brain tumor therapy, reducing side effects and improving drug bioavailability (27).
Nanoparticle-Drug Conjugates	Attaching drugs to nanoparticles for improved stability and targeted delivery to the brain in Alzheimer’s disease (28).	Utilizing targeted drug delivery for brain tumor treatment, delivering cytotoxic agents directly to tumor cells (29).

### 2.1 CNTs: characterization, types, synthesis

**Figure 2:**
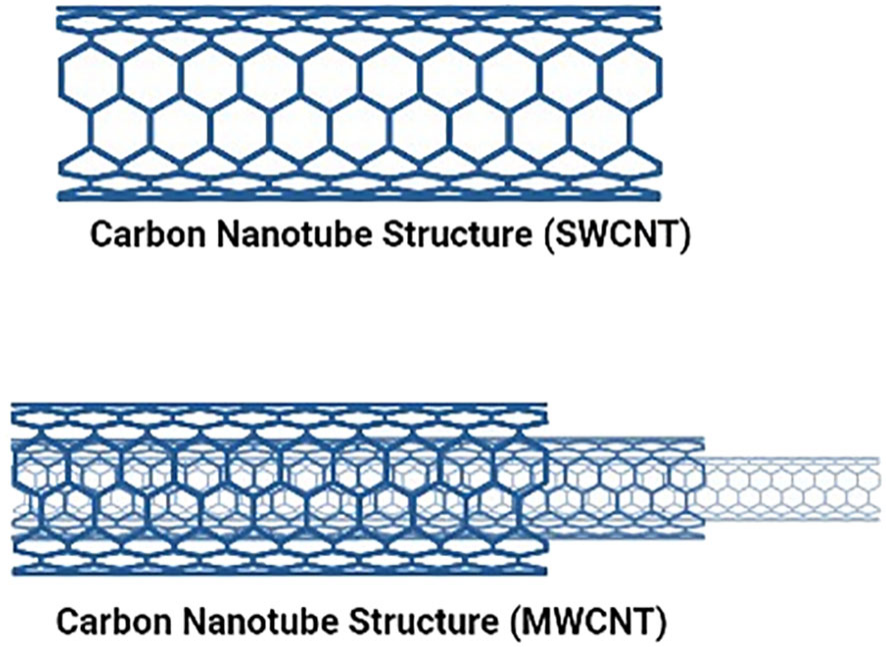
Shows the Single and multiwalled structures of Caron nanotubes.

CNTs discovered in 1991 (11), are a new allotrope of carbon that warrants special attention because of their inborn characteristics like their surface, form, and physical properties, which make them particularly ideal for therapeutic applications (30). A graphene sheet is folded into a cylindrical shape to create CNTs, which are tubular objects (31). Single-walled carbon nanotubes (SWCNTs), composed of one graphene sheet with a diameter between 0.4 and 40 nm, and multiwalled carbon nanotubes (MWCNTs), consisting of many layers forming cylinders with a distance of 0.35 nm between the concentric layers as shown in **Figure 2**. Typically, half-fullerene molecules shut nanotubes’ ends, while pentagonal defects serve as the tips. Furthermore, CNTs can be divided into three groups on how the sheets are revolved: zig-zag, chiral nanotubes, or armchair. Each carbon atom in CNTs is associated with an sp^2^ hybridization, giving them exceptional mechanical, optical, thermal, and electrical capabilities. One electron in the s orbital is promoted to one of the 2p atomic orbitals as part of the sp2 hybridization, a combination of one s and two p atomic orbitals. These two atomic orbitals combine to form three new, equally energetic hybrid orbitals. The energy of the hybrid orbitals is higher than the energy of the s orbital, lower than that of the p orbitals, but closer to the energy of the p orbitals. Trigonal structures are produced by the newly created hybrid orbitals, giving rise to a 120-degree molecular geometry. Therefore, sp2 hybridization makes stronger bonds. They are among the most reliable nanomaterials for potential uses in nanomedicine and nanoelectronics (11). Moreover, because of their capacity to cross cell membranes, these substances have been studied for their ability to generate ion transport channels, i.e., transporters for various drugs, biomolecules, and Deoxyribonucleic acid (DNA), and Ribonucleic acid (RNA). Additionally, because of their smaller internal size and unique electrical properties brought on by the curvature of their walls, CNTs have also been employed as nanoreactors (32). However, despite having a huge range of biomedical uses, carbon nanotubes possess two flaws that need to be addressed: their limited solubility in water and inherent toxicity induced by the metal catalyst residue left over from their production process.

Three methods are frequently utilized to create carbon nanotubes:

1. Chemical Vapor Deposition (CVD) method
2. Arc Discharge method
3. Laser Ablation method

**Figure 3:**
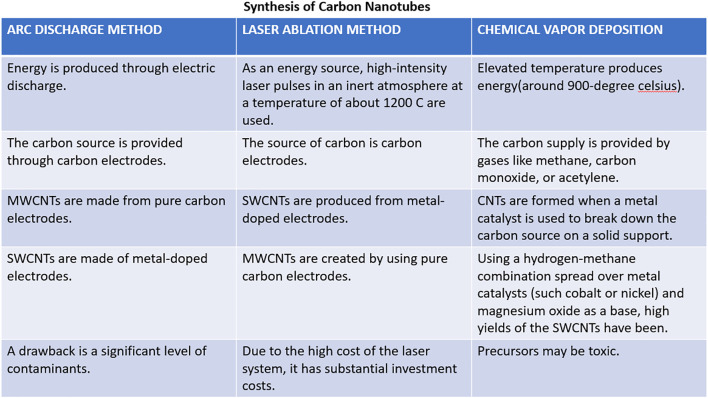
Common techniques for the generation of CNT.

These techniques are based on forming single or multiple carbon atoms that can merge to form carbon nanotubes. To synthesize carbon nanotubes, all processes require a source of energy and carbon (**Figure 3**). Carbon sources can be gases or carbon electrodes, and energy sources can include arc discharge, heat, or laser beams (33). Additionally, it is proposed that the CNT diameter is influenced by the dimensions of the metal catalyst’s particle; finer metal catalyst particle sizes are likely to produce SWCNTs with a narrower diameter, whilst bigger metal catalyst particle sizes seem to make MWCNTs with a broader diameter (34).

#### 2.1.1 Chemical vapor deposition method

The most popular technique for producing carbon nanotubes on a substantial scale is chemical vapor deposition. It uses hydrocarbons such as carbon monoxide, CH_4_, or acetylene, and elevated temperature gives the hydrocarbons enough energy to break down and generate carbon nanotubes. Commonly, SWCNTs can be created by chemical vapor deposition of acetylene utilizing a catalyst (iron or cobalt) supported by silica or zeolite; this leads to the production of large volumes of carbon nanotubes (34).

#### 2.1.2 Arc discharge method

The arc discharge method is the most popular and simple method for manufacturing CNTs, which has been used for many years to create fullerenes. Carbon electrodes are being employed as the source, and with the help of current (roughly within the range of 50 A to 100 A) and potential difference (nearly 20V), an electric discharge is generated, which acts as an energy source. It is considered that an electric discharge inside an inert environment at reduced pressure and with a catalyst such as nickel, iron, or cobalt creates enough high temperature to cause one of the carbon electrode surfaces to evaporate and create a tiny rod-shaped deposition of nanotubes on another electrode (35). This technique produces nanotubes with a diameter ranging between 0.6 nm - 1.2 nm (34).

#### 2.1.3 Laser ablation method

This approach uses carbon electrodes as the carbon and energy sources through laser pulses. The surface is uniformly vaporized by consecutive laser pulses, which also minimizes the quantity of carbon that has settled down as soot (36). The second pulse is believed to break up the bigger particles and the first laser pulse ablates smaller particles, subsequently developing into structures called nanotubes. Typically, transition metal catalysts are employed in this procedure, and the nanotubes produced with it are often achieved as rope-like structures with consistent diameters of around 10 nm to 20 nm, and the length of tubes is around 100 µm (37).

### 2.2 CNTs: holistic approach in managing AD and brain tumor

Since their discovery in the 1990s, the application of carbon nanotubes has been explored in several areas, including nanotechnology, industrial production, construction, electronics, wastewater management, and others (38–42). CNTs demonstrate a variety of distinctive attributes, including electrical and optical properties, a substantial surface area, and defined physicochemical characteristics. Solubility and toxicity limit the application of CNTs in biomedical sciences, but they can be reduced to a great extent with functionalization. The use of nano drug delivery in treating chronic neurological disorders results in neuroprotection. Nanomedicine encompasses various nanotechnology therapeutic applications (43). According to recent studies, significantly less nanomedicine has been used for treating neurodegenerative diseases compared to diseases like cancer and some infectious diseases (44). Although several studies have demonstrated that CNTs can efficiently permeate the BBB (45), the respective impermeability of the BBB is caused by tight connections created by endothelial cells of the brain’s capillaries. This causes some tiny and huge therapeutic molecules to not pass the BBB (46). The CNTs can penetrate the BBB through receptor-mediated pathways. CNTs have a massive surface area that allows loading and distributing high medication dosages to the therapeutic site. They also have inherent optical and thermal capabilities that could be used for photo-thermal and multimodal real-time tracking applications (45). The intrinsic barrier-crossing capacity of CNTs makes them ideal for use as nanocarriers for brain delivery, but because of unspecific bioaccumulation, this capacity is limited. As a result, choosing a particular brain target is crucial to enhance brain inflammation and minimizing side effects (45).

A study by Lohan et al. demonstrated that berberine (BRB) loaded MWCNTs with phospholipid and polysorbate coating effectively manage AD (47). In the SH-SY5Y cell lines, there was potential uptake of BRB-loaded MWCNT (47). Moreover, in rats, the enhanced performance was seen using the Morris Maze test. Also, the coating of phospholipid and polysorbate on MWCNT showed significant recovery in memory (47). The preservation of the usual biochemical amount in brain tissue established the ability of these coated MWCNTs to lessen β-amyloid-induced AD. The results show that polysorbate/phospholipid-coated MWCNTs of BRB have substantial potential for treating AD. A study by Mirali et al. showed that CNTs loaded with tacrine (Alzheimer’s drug) are safe for the specific delivery of drugs (36). The arrangement of Aβ (16-22) octamers was studied both in the presence and absence of SWCNT (48). It was observed that SWCNT could inhibit the Aβ (16-22) octamers and Aβ fibrillation (48). Furthermore, by correcting aberrant mTOR signaling activity and deficiencies in lysosomal proteolysis, SWNT restored healthy autophagy and facilitated the removal of autophagic substrates. These results suggest SWNT as a potential neuroprotective strategy for treating AD (49).

### 2.3 CNTs: holistic approach in managing brain tumor

**Figure 4:**
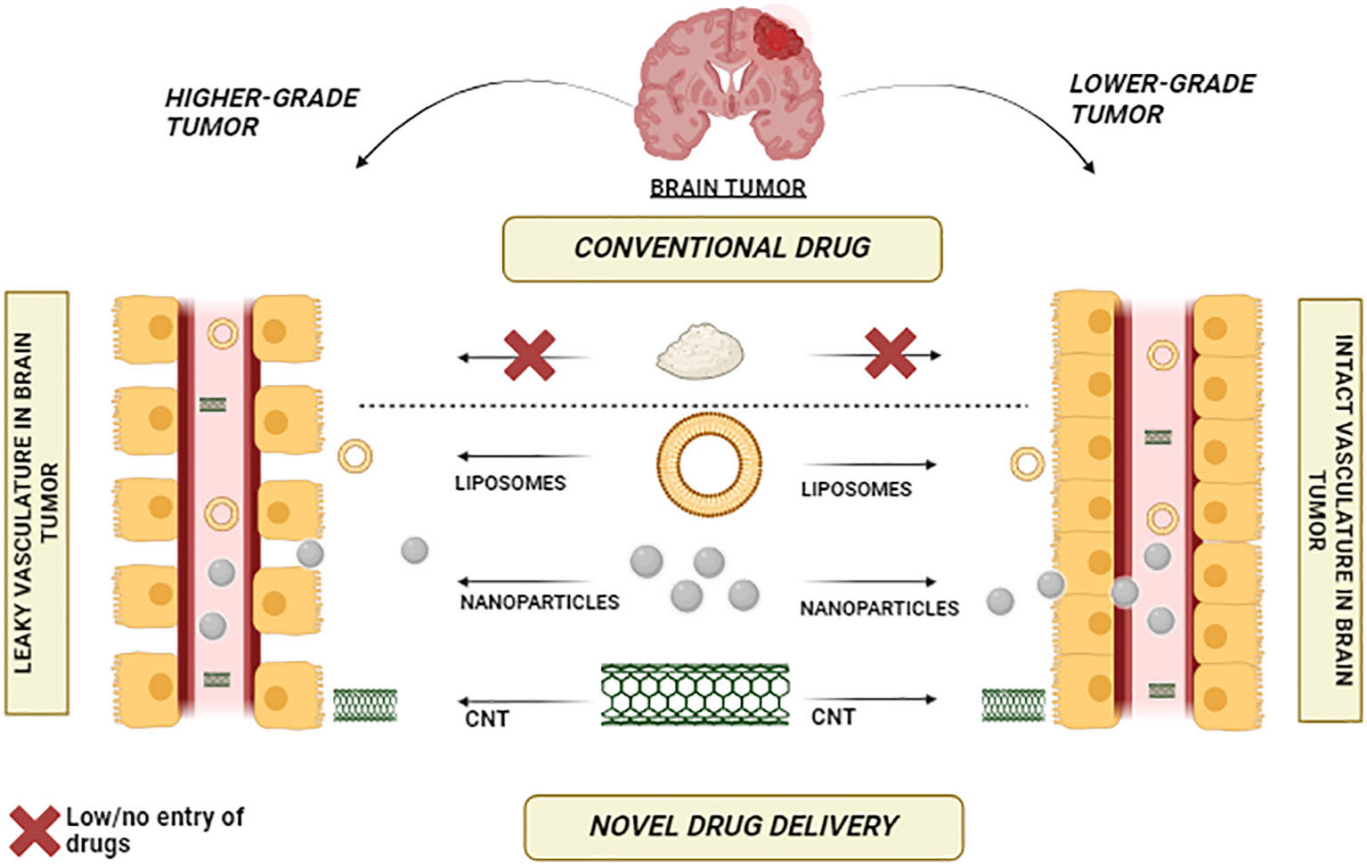
Bridging the Barrier – Nanoparticles, CNTs, and Liposomes Showcase Effective Blood-Brain Barrier Penetration.

A holistic approach to managing brain tumors involves the innovative utilization of Carbon Nanotubes (CNTs) across various facets of diagnosis and treatment. A comprehensive strategy emerges by leveraging CNTs’ unique properties, such as their exceptional surface area and ability to traverse the blood-brain barrier. Functionalized CNTs can be tailored to target and image brain tumor cells, enabling early and precise detection through advanced imaging techniques. **Figure 4** vividly illustrates the stark comparative distinction between conventional drugs and the remarkable efficacy of nanoparticles, carbon nanotubes (CNTs), and liposomes in traversing the formidable blood-brain barrier. While traditional drugs struggle to breach this barrier, the visual depiction showcases the extraordinary capability of nanoparticles, CNTs, and liposomes to overcome this challenge, opening new avenues for enhanced drug delivery to the brain.

Various studies have highlighted the significant role of CNTs in managing brain tumors. Glioma cells that cause brain tumor produce immunosuppressive compounds such as transforming growth factor-β, prostaglandins E, and interleukin (IL)-10, which could make them capable of staying away from the host’s immune system. Because of this, it is difficult to treat these tumors with conventional chemotherapy properly. To address this problem, Van Handel and Colleagues proposed an innovative immunotherapy strategy utilizing the multiwalled carbon nanotubes (MWCNTs), leveraging that macrophages preferentially absorb CNTs over glioma cells. The administration of MWCNTs led to heightened macrophage infiltration into glioma cells, resulting in a time- and dose-dependent increase in tumor cytokine levels, particularly IL-10. Notably, this treatment did not induce notable toxicity in either healthy mice or mice with tumors. These findings indicate that employing CNTs for immune modulation could be a promising approach for brain tumor therapy.

Indeed, the results imply that harnessing the immune-modulating characteristics of CNTs holds great promise as a strategy for addressing brain tumors. By bolstering the immune response against glioma cells, this approach offers a potential avenue for a more effective therapy for brain tumors.

Fan et al. (50) conducted a study investigating the systemic antitumor response when CpG was delivered intracerebrally with CNTs. They confirmed that this intracranial CNT–CpG therapy not only inhibited the growth of brain tumors but also subcutaneous melanomas in a melanoma mouse model. Based on their results, they suggested that intracerebral CNT–CpG therapy could be a viable treatment for gliomas and metastatic brain tumors.

In a similar study, Zhao et al. (51) examined using CNTs to deliver CpG in brain tumor models. The researchers observed that CNTs enhanced the uptake of CpG by tumor-associated phagocytic cells, leading to their activation both *in vitro* and *in vivo*. When a low dose of CNT–CpG complexes was administered via a single injection, it eradicated intracranial GL261 gliomas in half of the tumor-bearing mice by activating NK and CD8 cells. These findings demonstrated that CNTs facilitated improved CpG uptake into tumor-associated inflammatory cells without causing toxicity and resulted in a robust antitumor response.

## 3 Potential role of CNTs in the treatment of AD and brain tumors

### 3.1 Role of CNTs in drug, gene, and vaccine delivery for treatment of AD and brain tumor

The key challenges with present drug administration methods include lack of selectivity among normal and diseased tissues, metabolic elimination, failure to pass the cellular and blood-brain barrier (BBB), and drug degradation before reaching the target organs, which can drastically limit the overall therapeutic effects. As the outcome, ongoing research is focusing more on developing a mechanism for delivering drugs that is highly effective, intending to create tailored medicines using a carrier or vehicle. Targeted drug delivery can be accomplished using several approaches and routes, **Table 2**. For instance, several vectors, including nanocomposites, quantum dots, emulsions, dendrimers, and polymers, are used. These approaches, however, have a few drawbacks. For example, since the polymers in a polymer hydrogel formulation containing erythropoietin (EPO) are not biodegradable, surgical removal is necessary once the medicine has been delivered. CNTs can be used to solve these problems instead of polymers. Additionally, since CNTs are biodegradable, there is no need for surgical removal following drug administration. Due to their excellent drug-loading capacity, extensive surface area, remarkable mechanical strength, and adequate chemical stability, CNTs can be ideal nanocarriers for drug delivery (57).

**Table 2:** Shows different CNTs used for drug delivery for various diseases.

S.No.	Principle of study	Type of CNT	Disease	Result	Reference
1.	Functionalized single-walled CNTs were conjugated with CpG (oligodeoxynucleotides) (CNT–CpG).	SWCNT	Glioma	CNT-CpG enhanced CpG uptake, increased cytokine production, and eradicated gliomas in half of the mice, resulting in durable tumor-free remission and protection from tumor rechallenge.	(51)
2.	SWCNT–COOH Forming Supramolecular Complexes through π–π Stacking	SWCNT	Parkinson’s disease	SWCNT–COOH complexes with levodopa (LD), exhibited favorable sustained-release characteristics for over 20 hours, while the LD-loaded nanohybrid demonstrated pH-activated drug release and no compromise in cell viability in PC12 cell lines.	(52)
3.	MWCNTs-PEI-R-Nb was a targeted delivery system constructed using MWCNTs conjugated with polyethylenimine, ribavirin, and a PGNNV (virus)-specific nanobody.	MWCNT	Virus-induced CNS disease	MWCNTs-PEI-R-Nb showed targeted distribution, strong anti-PGNNV activity, and reduced mortality (27%) compared to the control group (100%) in infected zebrafish larvae.	(53)
4.	Chemically modified SWCNT with COOH and MADOPAR(Levapoda+benserazide)	SWCNT	Parkinson’s disease	Exhibits high adsorption efficiency, increased dispersibility in water, and improved safety, making it a promising drug carrier for benserazide and an effective treatment option for Parkinson’s disease.	(54)
5.	SWCNTs were carboxylated and loaded with Droxidopa (DOPA).	SWCNT	Parkinson’s disease	Droxidopa loading on carboxylated single-walled carbon nanotubes improves their dispersibility, enhances bioavailability, and reduces systemic toxicity.	(55)
6.	PEGylated oxidized multiwalled carbon nanotubes (O-MWNTs) were modified with angiopep-2 (O-MWNTs-PEG-ANG).	MWCNT	Glioma	DOX-O-MWNTs-PEG-ANG showed improved anti-glioma effects, good biocompatibility, and low toxicity, making it a promising carrier for brain tumor treatment.	(56)

Numerous anti-cancerous CNT-based drug delivery schemes work by incorporating a particular drug or gene into the tips and walls of CNTs that find cancer-specific cell surface receptors. This allows CNTs to pass through the membrane of a mammalian cell through main endocytosis, carrying therapeutic drugs/genes more effectively and reliably into cells that were not previously accessible. Functionalized CNTs have been evaluated as a vehicle for drug delivery for a range of antineoplastic drugs, including topoisomerase inhibitors (irinotecan, topotecan, doxorubicin (DOX), and epirubicin), platinum-based compounds (cisplatin, 5-fluorouracil, carboplatin), and anti-microtubule drugs (paclitaxel, docetaxel). Interestingly, Maleki et al. also evaluated N-isopropyl acrylamide carbon nanotube as a drug delivery structure through molecular dynamics simulation study using DOX loading (58). The interactions between the medicines and the carrier, hydrogen bonds, the gyration radius, and the radial distribution function are key notable elements for examining this carrier. Furthermore, by analyzing the DOX simulation studies, N-isopropyl acrylamide and carbon nanotubes can be used for drug delivery.

CNTs are also being researched as potential carriers for other diseases, including HIV, diabetes, and brain disorders (9, 59–61). Additionally, Yoosefian et al. conducted a molecular analysis study of the drug delivery system for an anti-Perkinson drug, Droxidopa, loaded with functionalized SWCNTs. The result of this simulation study showed improved bioavailability with reduced systemic toxicity (55).

Carbon nanotubes can function as an efficient vector system for the production and delivery of the therapeutic gene in the target tissues because nucleic acids and carbon nanotubes interact electrostatically to generate a variety of chemical complexes. This makes a variety of procedures involving gene therapy, vaccinations, and immuno-stimulating activities possible. (62). The delivery capability of CNTs is demonstrated by Cationic Polymer Brush-Modified Carbon Nanotubes for effective siRNA suppression of PD-L1 (Programmed death-ligand 1), enhanced biocompatibility, and cellular uptake in cancer immunotherapy (63). SWNTs demonstrate improved biocompatibility and stability as a delivery system. Chen et al. created SWNTs with amylose derivatives containing poly(L-lysine) dendrons and TNFα (ADP@SWNT/TNFα). *Invitro* and *in-vivo* studies were done on the complex’s aqueous dissipation stability, cytotoxicity, gene transfection potency, and photothermal effect. The findings suggested that ADP@SWNT/TNFα had a high potential for use in tumor therapy by being able to inhibit tumor growth and metastasis in both studies (64).

The carbon nanotube’s adjuvant property might be able to resolve the main obstacles associated with vaccine delivery. The fundamental idea behind using CNTs in vaccine delivery is to attach the antigen to them while maintaining its conformation, which will cause an antibody response with the appropriate specificity (65). The CNTs can activate several immune response-related genes, including NF- κB, ILs, and TNFα. This activation can lead to both acute and chronic immune responses. Acute inflammation occurs rapidly after exposure, while chronic activation can result from prolonged or repeated exposure. Chronic immune activation can contribute to sustained inflammation, oxidative stress, and neurodegenerative processes. Reactive oxygen species (ROS) and oxidative stress may be produced due to CNT-induced immune activation. The study by Mostovenkoet al. reveals that exposure to carbon nanomaterials can cause neurological changes reflected in the peptidomics profile of the cerebral spinal fluid (CSF). These changes resemble early-stage neurodegenerative disease, emphasizing the need to monitor CSF peptidomics markers for assessing neurotoxicity and identifying potential risks associated with carbon nanomaterial exposure. The duration, dose, and frequency of exposure determine the extent of immune activation and its long-term effects on the brain (66, 67). Further research is needed to understand the underlying mechanisms associated with this fully.

This inspired clinical research on CNT-based delivery systems to improve the body’s immune response to challenging diseases like cancer (68). Luna Labs recently created the “CNTVac” delivery platform, which uses short carbon nanotubes. Carbon nanotube size is carefully regulated to have an HIV-1 particle-like morphology and be effective at intranasal delivery of a wide variety of antigens. It has increased local IgA and systemic antibody IgG responses in mice and rabbits. In addition to acting as an effective delivery system, CNTVac lowers the number of lipids necessary for vaccine dosing, thereby removing potential side effects. The information points to a possible platform technology for the delivery of vaccines (69).

### 3.2 CNTs and regenerative mechanism via targeting amyloid beta plaques and neurofibrillary tangles

The utilization of carbon nanotubes in tissue engineering is yet another significant application. The primary goal of tissue engineering is to switch out unhealthy or eroded tissue with biological alternatives that can be repaired and maintain normal function. Whereas the study of self-healing, in which the body employs its systems occasionally with extra assistance from external biological material to regenerate cells and repair tissues and organs, is a component of regenerative medicine, which also encompasses tissue engineering (31). The 3D scaffold is applied in tissue engineering to keep the tissues’ mechanical integrity, flexibility, and cell-specific biochemical environment. CNT-based 3D scaffolds are better compared for use in composite scaffolds due to their superior mechanical attributes, which include high tensile strength and elastic moduli. In neurology, osteology, and cardiology fields, using CNTs as scaffolds for neural system rejuvenation is a promising advancement.

Researchers have demonstrated that MWCNTs can oxidize and aggregate enzymes like rhBMP-2 (Recombinant bone morphogenetic protein 2), promoting the stimulation of alkaline phosphatase and the genomes Cbfa1(Core-binding factor subunit alpha-1) and COLIA1 (Collagen type 1), which supports osteogenic discrepancy in cultured mesenchymal stem cells distinct from human adipose. MWCNTs can also control subsequent gene therapy reactions without adding exogenous signaling molecules or any other particular ligands, as evidenced by the frequent stimulation of *in-vivo* ectopic bone repair in mouse dorsal muscles. Therefore, the cultivation of renewable bone tissue is also made possible by this CNT material (70). Nanofiber scaffolds based on chitosan, polyvinyl alcohol, and carbon nanotube seem to be a favorable approach in cardiovascular tissue engineering (71).

A recent study by Li et al. raises the possibility of a new method for repairing transected peripheral nerves that combines electrical stimulation with an electric conductive CNT/sericin conduit. This CNT/sericin conduit has advantageous qualities like bio-compatibility, bio-degradability, porous microarchitecture, and appropriate swelling properties (72). According to a study by Xia et al., multivalent polyanion-dispersed CNTs can be used to create nano-structured fibrous scaffolds that can control the future of induced pluripotent stem cells (IPS). These hPGS (hyperbranched polyglycerol sulfate) CNTs have a distinct 1D morphology, are stable in both physiological medium and water, and have good biocompatibility compared to commercially available dispersed SDS (sodium dodecyl sulfonate) and PSS (poly sodium-styrene sulfonate). It was discovered that the suggested technique could act as a biocompatible program for encouraging IPS cells’ adhesion and growth. Additionally, it opens up a new method for creating composites with functionalized carbon nanomaterials to regenerate musculoskeletal and cardiovascular tissue (73).

In a recent article, functionalized single-walled carbon nanotubes (f-SWCNTs) were used to reinforce high molecular weight polyethylene (UHMWPE) material used to make unicompartmental knee implants. The surface biocompatibility of the prepared nanocomposite samples tested with human osteoblast cells leads to an improvement in cell viability with great cell differentiation and growth and affirms the effectiveness of the produced nanocomposite matter in the formation of artificial hip and knee inserts (74). The dispersibility of the CNTs was enhanced by using functionalized MWCNT (f-MWCNT). In the presence of human osteoblast cells, the manufacturing of silver-exchanged hydroxyapatite/functionalized multiwall carbon nanotube (Ag-HA/f-MWCNT) implants significantly inhibits proliferation and induces significant apoptosis (75).

### 3.3 CNTs for neuroprotection and neuroregeneration

Neuroprotection is the protection of neurons’ structure and functionality. This method is frequently selected for patients with CNS disorders such as stroke, brain injuries, and neurodegenerative diseases, including AD, to end or slow the loss of neurons. Despite varied signs and symptoms, the mechanisms causing neurodegeneration are the same (76). Using neuroprotective medications can shield neurons from neurodegeneration by diminishing neuro-inflammation and apoptotic pathways (77).

Nanotechnology has several benefits in managing CNS disorders; in the future, patients and doctors may have access to more therapeutic alternatives. It is crucial to connect the development of synthetic and characterization approaches in chemistry and materials science with the advancement of nano-engineered applications in the nervous system (78). Although there are many published studies and proven conclusions, there are few treatments for neurodegenerative diseases, and more research is required to understand the mechanisms underlying neurological disorders. Regrowth of axons and rearrangement of the neural circuitry are the two critical strategies for encouraging the self-repair of broken axonal connections in neurons. Thus, for successful regenerative engineering (neuroplasticity) as a therapeutic approach for managing AD, the preservation of neurons (neurorestorative), promotion of an environment favorable for the regrowth of the degenerated neurons, also known as neurogenesis, and reconnection of neuronal circuits, followed by advancement of the plasticity of neuronal tissue, are required (79). According to Lovat et al., using a pure MWNT substrate improved hippocampus neuron cell adhesion and dendritic elongation. The signal processing of neurons is better when the dendritic extension is increased (80). Similarly, Mazzatenta et al. demonstrated that pure SWNTs encourage the development of neural circuits and the growth of hippocampus neurons. The neural circuit expands noticeably as electrical signal transmission within the neuronal network increases ((81) Jan et al. evaluated the compatibility of CNT substrates with neural stem cells based on cell survival and the development of neuronal processes equivalent to those seen with the frequently used growth substrate poly-L-ornithine (NSCs) (82). The successful transport of NSCs to CNS injury sites and their differentiation into neurons served as evidence of the effectiveness of CNTs (82).

## 4 CNTs as a diagnostic tool for Alzheimer’s disease and brain tumor

### 4.1 CNTs in biosensors

Numerous groups of researchers have investigated CNTs as sensing elements for biosensors because of their robust electrical or optical properties, which are highly sensitive to be changed by exposure to biomolecules. The increased surface/volume ratio of CNTs allows the ultra-rapid detection of biological components even at lower concentrations. CNT-based biosensors possess better sensitivity, faster response times, higher stability, greater lifespans, and improved shelf life compared to most metal oxide or silicon-based commercially available sensors. Hence, CNT-based biosensors are acknowledged as a next-gen aspect of ultrasensitive biosensing systems (83).

Biosensors for glucose are essential. They monitor glucose levels, making them an excellent product for people with diabetes. Protein sensors, immuno-sensors, nucleic-acid sensors, and infection sensors are critical attributes of nanotube biosensors (84–87). Azimiet al. proposed a new technique for modifying straight lined-up carbon nanotube arrays (VACNTs) for targeted glucose detection. The constructed electrode worked well as a point-of-care (POC) biosensor to identify glucose in human blood plasma with a detection limit of 1.1 μM (88). Amidated MWCNTs coupled with gold nanocage decorated SPCE (screen-printed carbon electrode) were capable of ultrasensitive detection of MALAT1 (Metastasis Associated Lung Adenocarcinoma Transcript 1) biomarkers in non-small cell lung cancer. The formed biosensor depicted a broad linear range and low detection limit (42.8 fM) with sufficient selectivity and stability (89). A paper-based peptide-encapsulated SWCNTs dipstick optical biosensor has been advanced for protease observation. The feasibility of the sensor was demonstrated by detecting trypsin activity in a urine sample of a pancreatitis patient (90).

An electrochemical biosensor was created on a carbon nanotube field-effect transistor (CNT-FET) to detect SARS-CoV-2 S1 in saliva samples. The biosensor was generated using CNT printing on a Si/SiO2 surface and immobilized anti-SARS-CoV-2 S1. The presented biosensor achieves a diagnosis range of 0.1 fg mL-1 - 5.0 pg mL-1 and a detection limit of 4.12 fg mL-1 in 2-3 minutes. And hence, it can serve as an example for detecting SARS-CoV-2 S1 antigen (91).

### 4.2 CNTs in imaging

Utilizing CNTs allows for the enhancement of imaging features and functionality. Carbon nanotubes are very desirable in the area of biomedical imaging due to their optical, mechanical, physiochemical, and versatile photophysical properties. In nuclear imaging, Raman scattering, magnetic resonance, photoacoustic, optical detection, and fluorescent video imaging, CNTs can be exploited as imaging contrast agents (92). Semiconductive SWCNTs make remarkable fluorescent bioimaging sensors due to their near-infrared (NIR) fluorescence emission. A Fluorescence near-infrared (NIR) nanosensor based on SWCNTs was developed to visualize the release of the neurotransmitter serotonin from blood platelets in real-time. Such nano-sensors have great potential for defining how cells modulate chemical signals in space and time to convey information (93). Another real-time imaging strategy for tumor imaging has been proposed in which bioengineered small mussel adhesive proteins (MAPs) CNTs were used as tumor-targeted carriers. Brief CNT hybrid near-infrared fluorophores (NIRF) were deposited in the tumor cells with excellent targeting and clearance (94). Stronger NIR emission of SWCNTs in contrast with E11 release by radical polymer grafting on the SWCNT surface can be achieved by radical polymerization utilizing surfactant-dispersed SWCNTs (95).

Saghatchi et al. reported that the multiwalled carbon nanotubes equipped with magnetic Fe_3_O_4_ and gold NPs (mf-MWCNT/AuNPs) are favorable in comparison with factors in imaging by ultrasounds, CT scans, and MRI due to the beneficial qualities in radio-, thermo-, and imaging therapies. Moreover, mf-MWCNT/AuNPs is a strong candidate for use as a multi-modal instrument in cancer therapy (96).

## 5 Limitations of CNTs

### 5.1 Biocompatibility and biodegradation of CNTs

**Figure 5:**
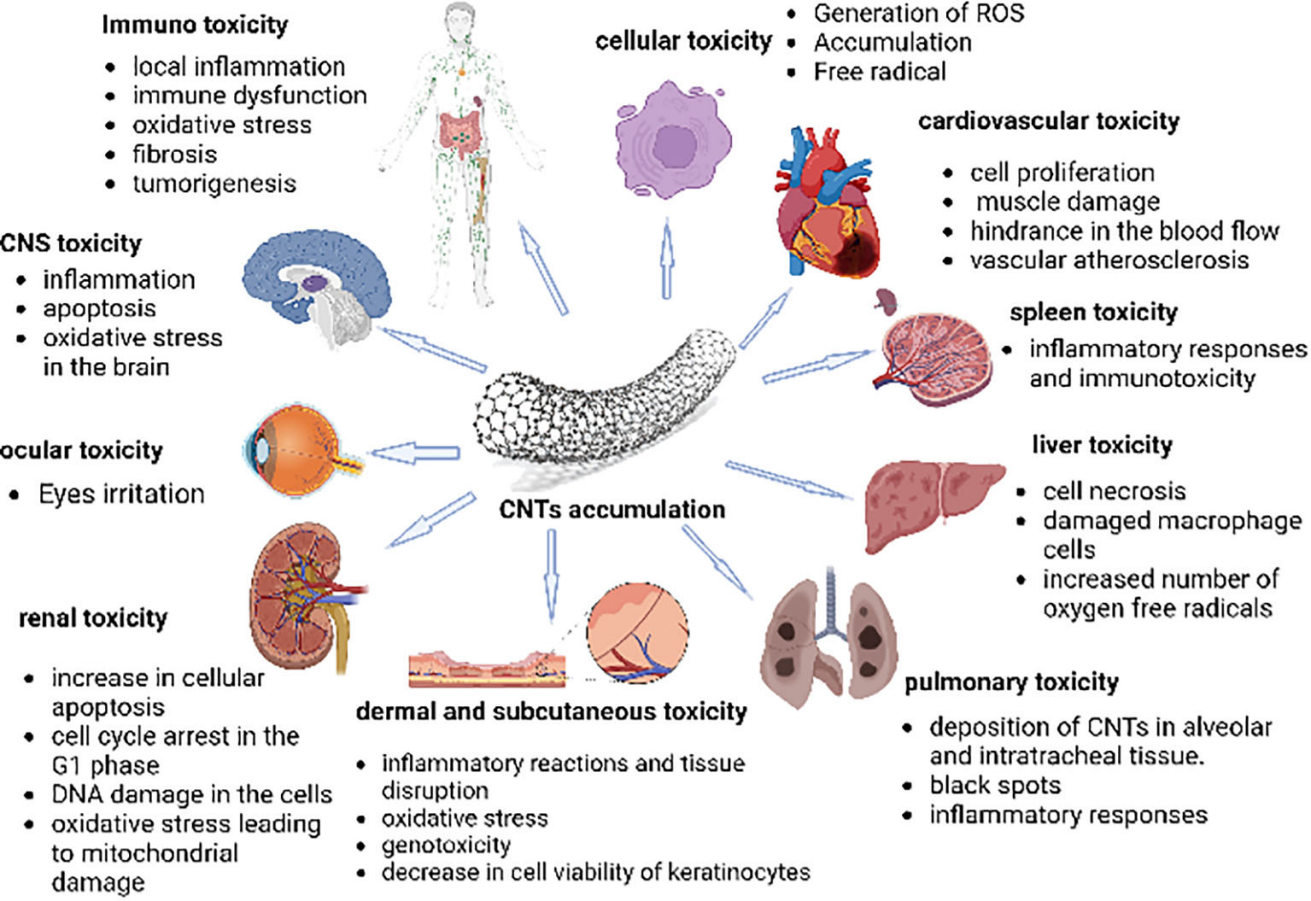
The figure represents the cytotoxic effects of CNTs on different body organs and immune systems. It depicts that penetration of carbon nanotubes leads to oxidative stress, increasing ROS (reactive oxygen species) levels in organs. The organs, such as the spleen, kidneys, and lungs, are most susceptible to the oxidative stress brought on by the generation of free radicals. Moreover, an inflammatory reaction is also observed towards the CNTs due to their immunogenicity, as these are considered foreign material, thus, eliciting a foreign body response (FBR) (99).

Due to their carcinogenicity and other harmful effects, nano-biomaterials may have adverse effects if they break down in the body. Nanoparticles that are not biodegradable can build up in tissues and be detrimental to people’s health. Studies on toxicity have been carried out to learn more about how to regulate the effects of CNT-based drugs on degradation (97). The degree of aggregation has also been shown to be one of the major factors affecting SWNT cytotoxicity. It has been demonstrated that human mesothelial cells are less damaging to CNT bundles than to CNT agglomerates. When dispersing nanotubes, cytotoxicity can be decreased by adding biocompatible surfactants such as poly-oxyethylene sorbitan mono-oleate 80 (PS80) (98).

(CNTs interact with different organs and bodily fluids. According to several experimental findings, CNTs are harmful to varying degrees in different tissues. Cells have been observed to experience oxidative stress when exposed to CNTs. Numerous studies have shown that the spleen, kidneys, and lungs are among the organs that are most susceptible to the oxidative stress brought on by the generation of free radicals. ROS are often produced as by-products of the metabolism of oxygen. Because CNTs induce oxidative stress within the cell, the quantity of ROS also rises. However, research into how they affect various organs is required before using them for medicinal purposes (99) **Figure 5**.

Numerous investigations into the biocompatibility and carcinogenicity of CNTs have been made. The results have been quite inconsistent; while some have claimed that the substance has great biocompatibility, others have claimed that it is carcinogenic (100). While more research has revealed that CNTs are potentially dangerous substances that may have adverse acute and long-term effects on a variety of living systems, some preliminary studies have revealed that CNTs are physiologically benign to particular cells, tissues, and organs like kidneys, stomach, bones, lymph nodes and lungs. In bone, CNTs may interfere in bone repair and new bone formation (101, 102). Whereas it seems that the biological effects of CNTs are sample-dependent and must be assessed individually. Therefore, more study is required to determine the nanotoxicity of CNTs.

### 5.2 Other challenges associated with CNTs are

- Lack of solubility in the majority of biologically compatible (aqueous-based) solvents.
- The creation of batch CNTs with the same chemically and structurally repeatable properties.
- Maintaining high quality and minimizing contaminants is challenging (103).

CNTs have a relatively low specific surface area and density and are often challenging to manufacture into thick electrodes using standard electrode preparation procedures, in contrast to advanced activated carbons(Acs), Carbide-derived carbons(CDCs) and zero -templated carbons (ZTCs) (104).

## 6 Future prospects

The potential applications of carbon nanotubes (CNTs) in the field of medicine and biotechnology are vast and promising. CNTs have already proven to be effective gene delivery and drug carriers for a wide range of medical purposes, including cancer treatment and anti-inflammatory therapies. The ability to transport various anticancer agents and therapeutic substances such as docetaxel (DTX), doxorubicin (DOX), methotrexate (MTX), paclitaxel (PTX), gemcitabine (GEM), and osteogenic dexamethasone (DEX) steroids via CNTs showcases their versatility.

Moreover, CNTs possess unique optical properties that make them valuable in phototherapy applications. Their simple surface functionalization has enabled their use as gene delivery vectors for addressing various diseases, including cancer, by transferring genes like plasmid DNA (PDNA), micro-RNA (miRNA), and small interfering RNA (siRNA). These advancements hold great promise for personalized medicine and targeted therapies.

However, it's important to acknowledge that concerns regarding CNT nanotoxicology and environmental impact remain. Since CNTs are not biodegradable, their long-term effects on the environment and human health need thorough investigation. Furthermore, despite decades of research and numerous in vivo and in vitro studies, widespread use of CNTs in medical applications awaits regulatory approval from agencies like the FDA (105).

## 7 Conclusion

Carbon nanotubes (CNTs) hold immense promise as versatile tools in the fields of medicine and biotechnology. Their ability to efficiently deliver genes and therapeutic agents to a wide range of cell types, including cancer cells, has the potential to revolutionize drug delivery and personalized medicine. Additionally, CNTs' unique optical and physiochemical properties make them valuable assets in imaging techniques and biosensors, enhancing their diagnostic capabilities.

## Author contributions

DE: Writing – original draft. GR: Supervision, Writing – original draft, Writing – review & editing. NK: Writing – original draft. PS: Writing – original draft. RJ: Writing – original draft. FK: Writing – original draft. BS: Writing – original draft. MK: Writing – review & editing. AB: Writing – review & editing. SF: Writing – review & editing.
